# The Effectiveness of Supervised Machine Learning in Screening and Diagnosing Voice Disorders: Systematic Review and Meta-analysis

**DOI:** 10.2196/38472

**Published:** 2022-10-14

**Authors:** Ghada Al-Hussain, Farag Shuweihdi, Haitham Alali, Mowafa Househ, Alaa Abd-alrazaq

**Affiliations:** 1 Department of Unified Health Record Lean for Business Services Riyadh Saudi Arabia; 2 Leeds Institute of Health Sciences, School of Medicine, University of Leads Leeds United Kingdom; 3 Health Management Department, Faculty of Medical and Health Sciences, Liwa College of Technology Abu Dhabi United Arab Emirates; 4 Division of Information and Computing Technology, College of Science and Engineering, Hamad Bin Khalifa University, Qatar Foundation Doha Qatar; 5 AI Center for Precision Health Weill Cornell Medicine Doha Qatar

**Keywords:** machine learning, voice disorders, systematic review, meta-analysis, diagnose, screening, mobile phone

## Abstract

**Background:**

When investigating voice disorders a series of processes are used when including voice screening and diagnosis. Both methods have limited standardized tests, which are affected by the clinician’s experience and subjective judgment. Machine learning (ML) algorithms have been used as an objective tool in screening or diagnosing voice disorders. However, the effectiveness of ML algorithms in assessing and diagnosing voice disorders has not received sufficient scholarly attention.

**Objective:**

This systematic review aimed to assess the effectiveness of ML algorithms in screening and diagnosing voice disorders.

**Methods:**

An electronic search was conducted in 5 databases. Studies that examined the performance (accuracy, sensitivity, and specificity) of any ML algorithm in detecting pathological voice samples were included. Two reviewers independently selected the studies, extracted data from the included studies, and assessed the risk of bias. The methodological quality of each study was assessed using the Quality Assessment of Diagnostic Accuracy Studies 2 tool via RevMan 5 software (Cochrane Library). The characteristics of studies, population, and index tests were extracted, and meta-analyses were conducted to pool the accuracy, sensitivity, and specificity of ML techniques. The issue of heterogeneity was addressed by discussing possible sources and excluding studies when necessary.

**Results:**

Of the 1409 records retrieved, 13 studies and 4079 participants were included in this review. A total of 13 ML techniques were used in the included studies, with the most common technique being least squares support vector machine. The pooled accuracy, sensitivity, and specificity of ML techniques in screening voice disorders were 93%, 96%, and 93%, respectively. Least squares support vector machine had the highest accuracy (99%), while the K-nearest neighbor algorithm had the highest sensitivity (98%) and specificity (98%). Quadric discriminant analysis achieved the lowest accuracy (91%), sensitivity (89%), and specificity (89%).

**Conclusions:**

ML showed promising findings in the screening of voice disorders. However, the findings were not conclusive in diagnosing voice disorders owing to the limited number of studies that used ML for diagnostic purposes; thus, more investigations are needed. While it might not be possible to use ML alone as a substitute for current diagnostic tools, it may be used as a decision support tool for clinicians to assess their patients, which could improve the management process for assessment.

**Trial Registration:**

PROSPERO CRD42020214438; https://www.crd.york.ac.uk/prospero/display_record.php?RecordID=214438

## Introduction

### Background

Voice disorders are abnormalities in voice production that could be due to lesions or abnormal modifications in the structure of vocal folds [1]. In 2019, it was estimated that 16.9% of the population in Sweden had voice disorders [2], and in 2014, it was found that 1 in 13 adults in the United States develops voice disorders every year [3]. This led to a loss of US $845 million in the United States owing to missed working days among employees with voice disorders [4,5]. At the individual level, voice disorders can severely affect a patient’s social life and mental health compared with other chronic disorders such as back pain [6]. Thus, 4.3% of the patients with voice disorders reported that they were unable to do certain job-related tasks due to the disorder [7]; this especially affects professions that have a high demand on the voice, for instance, teachers [8], singers, or telephone operators [9]. Therefore, screening or diagnosing voice disorders is essential to detect other related health conditions such as laryngeal lesions that could be a symptom of cancer [10]; thus, the diagnosis should be made as soon as possible [11,12].

Diagnosing and screening voice disorders involve auditory-perceptual and instrumental assessments. The auditory-perceptual assessment is carried out by a qualified speech and language therapist (SLT); in this assessment, the SLT determines the quality of patients’ voice by listening to their sustained vowel production; for example, the, aa, or sound or continuous speech [13,14]. Furthermore, the instrumental assessment involves laryngeal imaging to examine the structure and function of vocal cords while the patient produces a vowel sound; other techniques are also used including video laryngeoendoscopy and video laryngostroboscopy examinations. In addition, acoustic instruments were used to analyze acoustic features (frequency, pitch, volume, and quality of sound) of voice samples of patients to assess voice disorders by using computer software [13-15]. Although the aforementioned assessments are recognized by the American Speech-Language-Hearing Association [13] and American Academy of Otolaryngology-Head and Neck Surgery [16], there is still a lack of standardized methods and guidelines to regulate these or other assessments [17]. Therefore, several limitations may pose a risk to the current assessment [18,19]. Although each case is evaluated objectively (via instrumental techniques, eg, stroboscopy), these objective tests include acoustic and visual imaging and videos; the acoustic techniques reveal the speech characteristics of the patient’s speech sample, specifically, the frequency, intensity, loudness, and pitch, to give the clinician insight into other indicators such as the patient’s rate of speech or voice; for example, the voice may be breathy or tremored [18]. Although these instrumental methods enable clinicians to perform objective tests, the validity of the tests largely depends on the auditory-perceptual skills of the clinician [18]. This is because the clinician first assesses the instrumental management or the patients’ pathway and then chooses the type of instrumental assessment to be used. Naturally, any mistake in the auditory-perceptual assessment would affect the instrumental management, and thus, the whole management of the case; such subjective judgment might not be reliable as it relies on the clinician’s skills and experience [18]. As the condition of each diagnosis or screening and the level of experience differ in each case, severe cases might be easier to diagnose or screen than mild cases; therefore, the experience of the SLT and the reliability of their judgment on each patient’s condition differ, and low interrater correlations may occur (<0.9) [19]. Moreover, the agreement between experienced and inexperienced SLTs was found to be <75%, making the experience an essential part of the diagnosis or screening [20].

Machine learning (ML) was introduced for speech sounds in the early 1980s [21]. ML can be performed automatically by analyzing acoustic features either from voice recordings samples that are previously stored in a database such as the Massachusetts Eye and Ear Infirmary (MEEI), which are databases that stores a recordings of voice samples from patients in clinical environments, these recordings either recorded patients’ voices while pronouncing vowels such as in MEEI [22] or continuous speech, or phrases such as in the Saarbruecken Voice Database [23]. ML is also used to analyze patients in the clinic by recording their voices via a microphone [1,21,24]. ML was applied either as a differential diagnosis for s, which involves diagnosing the voice sample as 1 of 2 diseases (voice disorders a or voice disorders b), or for screening different voice samples as either healthy or pathological voice. This method has been used to improve the diagnosis and screening process to be more objective. ML involves 2 different models: classification (supervised learning) and clustering or categorization (unsupervised learning) [25]. In the unsupervised model, the algorithm categorizes and identifies relationships within a data set [26]. By contrast, classification is a prediction model that defines labels, for example, disease or not disease, in clinical diagnosis [26], making it more common in diagnosing [27].

### Research Problem and Aim

Although several studies have investigated the effectiveness of ML algorithms in detecting and diagnosing voice disorders, to the best of our knowledge, only 1 review attempted to summarize the evidence resulting from these studies [27]. However, there are several limitations in the review, including the following: it did not exclude studies that did not validate their ML outcomes by using validation techniques; it included studies that relied on scientific but not technical or objective solutions, and they relied on subjective assessment only; and it did not assess the included studies against any risk of bias assessment. Accordingly, this systematic review aimed to assess the effectiveness of supervised ML algorithms in screening and diagnosing voice disorders. Thus, only supervised ML techniques were considered because supervised ML algorithms are more commonly used for diagnosing and detecting disorders.

## Methods

This systematic review followed the Cochrane Library’s systematic reviews for diagnostic test accuracy (DTA) guidelines [28] to meet the objectives of this review. The protocol for this review was registered with PROSPERO (CRD42020214438).

### Search Strategy

#### Search Sources

The following 5 databases were searched on June 24, 2021: MEDLINE (via Ovid), Embase, Scopus, Web of Science, and ACM Digital Library. No language limitations were applied, and non-English articles were translated to check their applicability to the review. The retrieved references were exported and managed using EndNote 9.

#### Search Terms

A total of 2 groups of keywords were used to search the databases: one group representing the target diagnosis (ie, voice disorders) and the other group representing the intervention of interest (ie, ML algorithms). The terms were derived from ML and speech therapy experts. Medical Subject Headings were also included to maximize the sensitivity of the search in MEDLINE and Embase. The detailed search strategy that was applied to MEDLINE and Web of Science is shown in Multimedia Appendixes 1 and 2, respectively.

### Eligibility Criteria

#### Inclusion Criteria

The population of interest in this review included patients diagnosed with a voice disorder. No restrictions were applied to the type of population characteristics (eg, age, gender, and ethnicity). With regard to index tests, we focused on supervised ML techniques (classification) that were used to screen or diagnose voice disorders in binary outcomes (eg, pathological voice vs healthy voice or voice disorder a vs b) by using voice samples collected in a controlled environment (eg, speech laboratories, hospitals, clinics, and databases). The reference standards of interest in this review are instrumental assessment and auditory-perceptual assessment, as both follow the recommendations of the American Speech-Language-Hearing Association [17] and American Academy of Otolaryngology [16]. To be included in this review, studies had to assess the diagnostic performance of ML algorithms by using at least one of the following outcomes: accuracy, sensitivity, and specificity. We included only peer-reviewed articles and empirical studies regardless of their study design. No restrictions were applied on the country of publication, year of publication, or language of publication.

#### Exclusion Criteria

We excluded studies that relied on clinicians’ judgments only without using any instrumental tools to ensure the validity and reliability of the review, as relying on subjective assessment may be affected by the clinician’s level of experience. Unsupervised ML methods were excluded. Conference papers, reviews, reports, editorials, ongoing studies, non–peer-reviewed articles, studies that assessed accuracy only, and those that did not assess sensitivity and specificity were also excluded.

### Study Selection

Study selection was first conducted by screening the titles and abstracts of the retrieved studies. Although we excluded studies whose titles and abstracts did not meet any of the eligibility criteria, all studies that met the eligibility criteria or were unclear owing to a lack of information in their titles and abstracts were retained. We then read the full texts of the studies that remained after the title and abstract screening to assess their eligibility for this review. The study selection process was performed by 2 reviewers.

### Data Extraction

The 2 reviewers created a data extraction form (Multimedia Appendix 3) and extracted the data from each included study. If a study did not report a required piece of information, we contacted the corresponding authors to obtain any missing information. If the corresponding authors did not reply within 2 weeks, we sent 2 reminders. If we did not receive a reply after 2 weeks of the second reminder, the missing piece of information was referred to as *n/a: not applicable* data were extracted in an Excel spreadsheet.

### Evaluation of Methodological Quality

The risk of bias in the included studies was assessed using a revised tool for the Quality Assessment of Diagnostic Accuracy Studies (QUADAS)-2 [29], which is highly recommended by the Cochrane Collaboration [30]. QUADAS-2 assessed the risk of bias in 4 domains in the included studies: patient selection, index test, reference standards, and flow and timing (Multimedia Appendices 4-7). Furthermore, QUADAS-2 appraised the applicability of the included studies to this review in terms of 3 domains: patient selection, index test, and reference standards. QUADAS-2 was modified to fit this review (Multimedia Appendix 8). The 2 reviewers assessed the methodological quality of all included studies by using Review Manager (RevMan version 5.4).

### Data Synthesis and Analysis

Narrative and quantitative syntheses were conducted to analyze the outcome of each ML technique (accuracy, sensitivity, and specificity). If >1 study used the same ML technique, and the difference between the outcomes was not significant (<5%), the best outcome was considered in the meta-analysis. All outcomes are presented in the extraction table (Multimedia Appendix 3). In addition, if a study used voice samples from 2 different databases, each sample was included to account for the sample size (referred to as sample A and sample B in the forest plot).

The accuracy, sensitivity, and specificity of ML methods extracted from the eligible studies were analyzed using the random effect proportional meta-analysis to estimate a pooled proportion and 95% CI, which are based on the Wilson score [31] procedures. To stabilize the variances, the pooled estimate was calculated using the Freeman-Tukey double arcsine transformation [32], and heterogeneity was calculated using the *I*^2^ measure [33]. A value of ≤50% is considered low, 51% to 75% moderate, and ≥76% high [33]. All results were plotted and presented in a forest plot. Studies were included in the meta-analysis if their scope of using ML was for screening. Statistical software STATA 16 was used to perform random effects meta-analyses.

## Results

### Search Results

**Figure 1:**
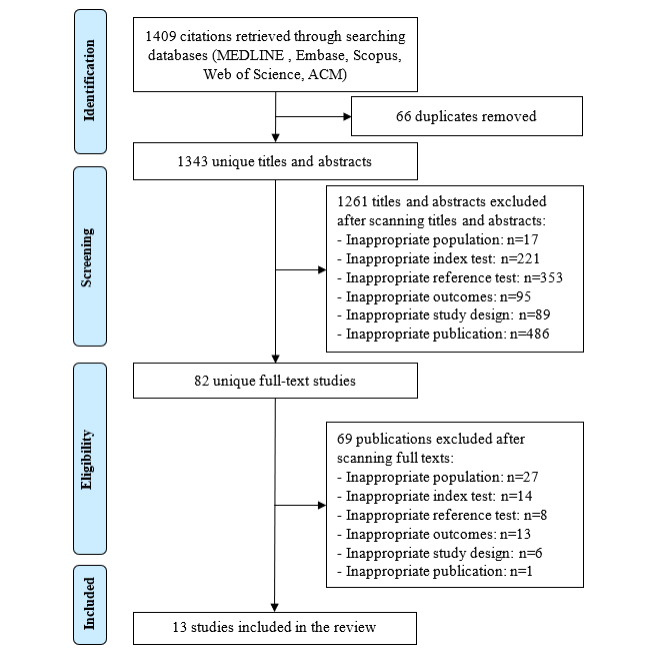
Flowchart of the study selection process.

As presented in Figure 1, a total of 1409 hits were identified by searching the 5 databases. No additional records were obtained from different resources. After removing duplicates, 95.31% (1343/1409) of articles were left. After scanning the titles and abstracts, 93.89% (1261/1343) of records were excluded, leaving 82 (6.11%) records for full-text reading. We excluded further 84% (69/82) of articles after full-text reading; therefore, only 16% (13/82) of studies were included in this review [34-46].

### Study Characteristics

#### Study Metadata

As shown in Table 1, the 13 included studies were conducted between 2000 and 2020. However, most of the studies (11/13, 85%) were conducted between 2010 and 2020. The year that witnessed the largest number of studies (3/13, 23%) was 2016. The included studies were conducted in 12 different countries, and approximately 30% (4/13) of them were conducted in Iran. All the studies were observational studies, peer-reviewed articles, and written in English.

**Table 1:** Metadata of the included studies.

Study	Year	Country	Publication language
Akbari and Arjmandi [34]	2015	Iran	English
Arias-Londoño et al [35]	2011	Greece	English
Arjmandi and Pooyan [36]	2012	Iran	English
Arjmandi et al [37]	2011	Iran	English
Cordeiro et al [38]	2017	Portugal	English
Ghasemzadeh et al [39]	2015	Iran	English
Godino-Llorente and Gómez-Vilda [40]	2004	Spain	English
Hadjitodorov et al [41]	2000	Bulgaria and France	English
Hariharan et al [42]	2014	Turkey	English
Lopes et al [43]	2017	Brazil	English
Mohmmad et al [44]	2020	Saudi Arabia and Malaysia	English
Souissi and Cherif [45]	2016	Tunis	English
Wang et al [46]	2011	China	English

#### Participants or Sample Characteristics

The number of participants or voice samples ranged from 40 to 960, with a total of 4019 and an average of 309 (Table 2). The included studies collected data from 6 different sources. The MEEI database was the most commonly used database among the included studies (9/13, 69%). Voice samples were collected from male and females and intersex participants in most included studies (12/13, 92%); however, 8% (1/13) of studies used voice samples from female participants only [43]. Participants’ ages in the included studies ranged from 13 to 86 years, with an average age of 45 years (mean 46, SD 29.5 years).

**Table 2:** Characteristics of participants or sample.

Study	Voice sample size, n	Age (years), range	Male (%)	Setting or database	Database accessibility
Akbari and Arjmandi [34]	293	13-82	40	MEEI^a^ database	Private
Arias-Londoño et al [35]	628	19-70	—^b^	MEEI and UPM^c^ databases	Private
Arjmandi and Pooyan [36]	120	18-86	56	MEEI database	Private
Arjmandi et al [37]	100	16-85	67	MEEI database	Private
Cordeiro et al [38]	154	—	34	MEEI database	Private
Ghasemzadeh et al [39]	393	—	—	MEEI database	Private
Godino-Llorente and Gómez-Vilda [40]	135	—	—	MEEI database	Private
Hadjitodorov et al [41]	400	—	—	Phoniatric Department of the University Hospital in Sofia	Private
Hariharan et al [42]	274	20-68	—	MEEI and MAPACI databases	Private
Lopes et al [43]	279	18-65	0	Voice laboratory	Private
Mohmmad et al [44]	960	—	—	SVD^d^	Private
Souissi and Cherif [45]	120	—	—	SVD	Private
Wang et al [46]	226	26-58	—	MEEI database	Private

^a^MEEI: Massachusetts Eye and Ear Infirmary.

^b^Not available.

^c^UPM: Universidad Autónoma de Madrid.

^d^SVD: Saarbruecken Voice Database.

#### Index Test Characteristics

The included studies used 12 ML algorithms (Table 3). Least-squares support-vector machines (LS-SVMs) were the most used algorithms across studies (9/13, 69%), followed by quadratic discriminant analysis (QDA) (3/13, 23%) and K-nearest neighbor (K-NN) (4/13, 31%). The feature-extraction technique was reported in 85% (11/13) of studies. While 61% (8/13) of studies extracted short-term features (eg, mel frequency cepstral coefficients), 23% (3/13) extracted long-term features (eg, jitter and shimmer and fundamental frequency). A total of 3 feature reduction techniques were used in the included studies; linear discriminant analysis was the most used technique (4/13, 31%), and training-test split validation was the most prominent technique used in the included studies (10/13, 77%), followed by cross-validation technique (4/13, 31%).

**Table 3:** Index test characteristics.

Study	Machine learning method	Feature extraction	Feature reduction	Validation
Akbari and Arjmandi [34]	LS-SVM^a^	Mean, variance, skewness, kurtosis of coefficient, wavelet subband coefficients	Linear prediction analysis and LDA^b^	70% training and 30% 0% testing
Arias-Londoño et al [35]	LS-SVM	12 MFCC^c^ and MSMR^d^	MSMR and LS-SVM	75% training and 25% testing (cross-validation–test split validation)
Arjmandi and Pooyan [36]	QDA^e^, NMC^f^, K-NN^g^, LS-SVM, ML-NN^h^, and PC^i^	PCA^j^ and LDA; feature selection: IFS^k^, FFS^l^, BFS^m^, and BBFS^n^	PCA and LDA	70% training and 30% validation
Arjmandi et al [37]	QDA, NMC, PC, K-NN, LS-SVM, and ML-NN	Fundamental frequency (average, high, and low variation), STD^o^, PFR^p^, jitter, shimmer, RAP^q^, PPQ^r^, smoothed PPQ, vAm^s^, NHR^t^, VTI^u^, SPI^v^, FTRI^w^, ATRI^x^, Tsam^y^, T0^z^, shimmer in dB, DVB^aa^, DSH^ab^, DUV^ac^, NVB^ad^, NSH^ae^, and total number of segments pitch period during the period-to-period pitch extraction	PCA and LDA	70% training and 30% testing
Cordeiro et al [38]	SVM and DA^af^	MFCCs, line spectral frequencies, and delta-MFCC	N/A^ag^	75% training and 25% testing (k-fold cross-validation method, k=4; training-test split validation)
Ghasemzadeh et al [39]	ANN^a^^h^ and LS-SVM	False neighbor fraction and mutual information	LDA and LS-SVM	70% training and 30% testing using cross-validation
Godino-Llorente and Gómez-Vilda [40]	LVQ^a^^i^	MFCC coefficient, energy, and first and second temporal derivatives	MFCC	70% training and 30% test split validation
Hadjitodorov et al [41]	K-NN	Pitch period (To), PPQ, APQ^aj^, STAB^a^^k^, the degree of the dissimilarity of the shape [47] of the pitch pulses, LHER^a^^l^, NHR, HNR^am^, and energy in the pitch impulse-incepstra	LDA	Training-test split validation stage (200 phonation); testing (200 phonation)
Hariharan et al [42]	K-NN, LS-SVM, and GRNN^an^	5 level WPT^ao^ decomposition	N/A	70% training and 30% testing using conventional validation and cross-validation
Lopes et al [43]	QDA	F0 measurements (mean and SD, jitter, shimmer, and GNE^ap^)	N/A	Cross-validation
Mohmmad et al [44]	CNN^aq^	Octaves and its first and second derivatives	N/A	10-fold cross-validation
Souissi and Cherif [45]	LS-SVM and ANN	MFCC and first and second derivatives	MFCC, LDA, and delta	70% training; and 30% testing
Wang et al [46]	LS-SVM and GMM^a^^r^	36 dimensional MFCC parameters with 1 derivative were calculated every frame of 18-mel-cepstral coefficient	8, 16, and 32 mixture	10-fold cross-validation

^a^LS-SVM: least-squares support-vector machine.

^b^LDA: linear discriminant analysis.

^c^MFCC: mel frequency cepstral coefficient.

^d^MSMR: modulation spectra minimum redundancy.

^e^QDA: quadric discriminant analysis.

^f^NMC: neuromorphic computing.

^g^K-NN: K-nearest neighbor.

^h^ML-NN: multilayer neural network.

^i^PC: Parzen classifier.

^j^PCA: principal component analysis.

^k^IFS: individual feature selection.

^l^FFS: forward feature selection.

^m^BFS: backward feature selection.

^n^BBFS: branch-and-bound feature selection.

^o^STD: SD of fundamental frequency.

^p^PFR: phonatory fundamental frequency.

^q^RAP: relative average perturbation.

^r^PPQ: pitch perturbation quotient.

^s^vAm: peak amplitude variation.

^t^NHR: noise-to-harmonic ratio.

^u^VTI: voice turbulence index.

^v^SPI: soft phonation index.

^w^FTRI: Fo-tremor intensity index.

^x^ATRI: amplitude tremor intensity index.

^y^Tsam: length in seconds of analyzed voice data sample.

^z^T0: period of the average glottal period.

^aa^DVB: degree of voice breaks.

^ab^DSH: degree of subharmonic.

^ac^DUV: degree of voicelessness.

^ad^NVB: number of voice breaks.

^ae^NSH: number of subharmonic segments.

^af^DA: Discriminant analysis.

^ag^N/A: not applicable.

^ah^ANN: artificial neural network.

^ai^LVQ: learning vector quantization.

^aj^APQ: amplitude of the pitch pules.

^ak^STAB: stability of the t0 generation.

^al^LHER: low-high energy ratio.

^am^HNR: harmonics noise ratio.

^an^GRNN: general regression neural network.

^ao^WPT: wavelet packet transform.

^ap^GNE: glottal to noise excitation.

^aq^CNN: conventional neural network.

^ar^GMM: Gaussian mixture model.

### Quality Assessment Results

#### Risk of Bias

In the patient selection domain, only 38% (5/13) of studies were judged to have a low risk of bias in patient sampling, as they used an appropriate sampling process to select voice samples (Multimedia Appendix 9). The risk of bias in index tests was rated as high in all included studies owing to the nature of the supervised ML tests, and their results were interpreted with prior knowledge of the results of the reference standard test. Owing to the subjective nature of voice assessment, it was not clear whether the reference standard correctly classified the patients. This led to an unclear risk of bias in the reference standard domain in all studies although the reference standard was used before the index test, and the findings were not affected by the findings of the index test. Patient flow and timing were poorly reported in almost all the studies (12/13, 92%). Thus, these studies were judged to pose an unclear risk of bias in terms of patient flow and timing. Multimedia Appendix 9 shows the QUADAS-2 tool risk of bias judgment in each included study across all 3 domains as well as applicability concerns for each study.

#### Applicability Concerns

There are no applicability concerns regarding how patients were selected in all included studies, as the patients’ characteristics and the condition and setting of each test match the review question and criteria (Multimedia Appendix 9). Similarly, all included studies were judged to have low applicability concern in the index test as the ML algorithms method in the included studies matched the review definition of ML. However, the applicability concern in the reference standard was rated as unclear in 84% (11/13) of studies, as the voice samples in those studies were collected from databases, and the detailed diagnosis process of each voice sample was not described.

### Performance of ML Algorithms

#### Diagnosing Voice Disorders

Only 8% (1/13) of studies used the QDA algorithm to differentiate between 2 [43]. As shown in Table 4, the accuracy, sensitivity, and specificity of the QDA ranged from 70% to 77%, 20% to 65%, and 74.76% to 95%, respectively. See the following section for a description of how QDA was used as a screening tool. For breakdown of the diagnostic findings, please refer to Multimedia Appendix 10.

**Table 4:** The performance of machine learning in diagnosing voice disorders.

Algorithm	Tested diseases	Accuracy (%)	Sensitivity (%)	Specificity (%)	Study
QDA^a^	Vocal polyps vs healthy	70.56	50	74.76	Lopes et al [43]
QDA	Vocal cyst vs healthy	72.67	60.83	78.1	Lopes et al [43]
QDA	Unilateral VF^b^ paralysis or healthy	79.82	20	92.38	Lopes et al [43]
QDA	Middle-posterior triangular gap vs healthy	71.11	45	80.43	Lopes et al [43]
QDA	Sulcus vocalis vs healthy	78.75	50	83.33	Lopes et al [43]
QDA	VDDGER^c^ vs healthy	72.44	33.33	90.71	Lopes et al [43]
QDA	Vocal nodules vs unilateral VF paralysis	76.61	20	88.57	Lopes et al [43]
QDA	Vocal nodules vs sulcus vocalis	72.68	50	75.95	Lopes et al [43]
QDA	Vocal nodules vs VDDGER	71	33.33	89.05	Lopes et al [43]
QDA	Vocal nodules vs sulcus vocalis	70	30	95	Lopes et al [43]
QDA	Vocal polyp vs healthy	75.14	65	78.33	Lopes et al [43]
QDA	Vocal cyst vs healthy	73.22	62.5	78.57	Lopes et al [43]

^a^QDA: quadratic discriminant analysis.

^b^VF: vocal fold.

^c^VDDGER: voice disorder due to gastroesophageal reflux.

#### Screening Voice Disorders

Of the 13 included studies in the systematic review, 10 (77%) were included in the meta-analysis and 3 (23%) were excluded [39,40,44,46]. Of the 10 studies, 2 (20%) examined ML techniques by using 2 different databases: Arias-Londoño et al [35] (MEEI and Universidad Autónoma de Madrid [UPM] databases) and Hariharan et al [42] (MEEIEMPACI) Accordingly, the performance of ML techniques in these databases was included in the meta-analysis. More information about the performance in screening can be found in (Multimedia Appendix 11).

#### Accuracy

**Figure 2:**
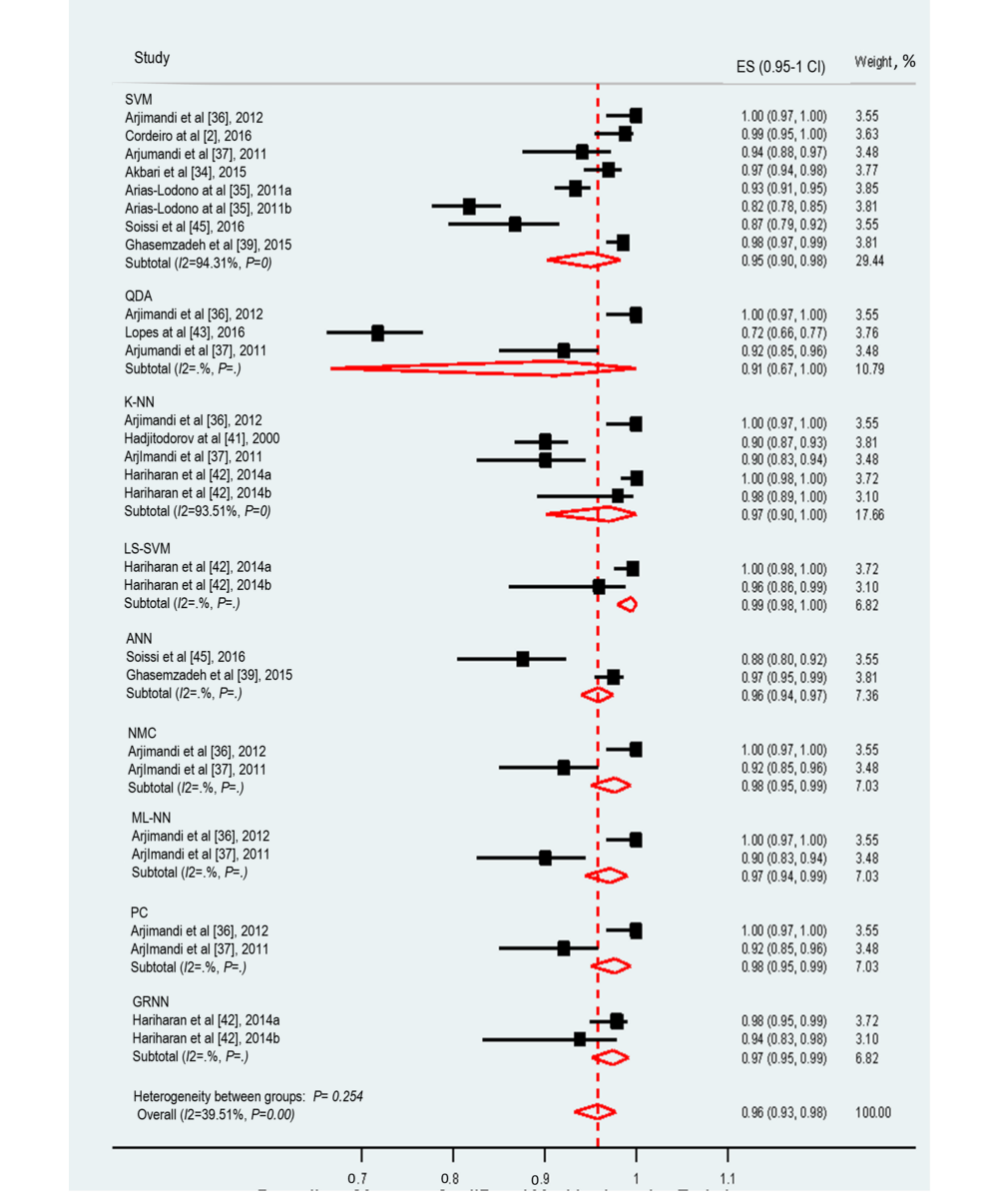
The forest plot shows the accuracy of machine learning algorithms in voice disorder screening. ANN: artificial neural network; GRNN: general regression neural network; K-NN: K-nearest neighbor; LS-SVM: least-squares support-vector machine; ML-NN: multilayer neural network; NMC: neuromorphic computing; PC: parzan Classifier; QDA: quadratic discriminant analysis; SVM: support vector machine.

The accuracy of ML techniques in assessing voice disorders was reported in 77% (10/13) of studies. These studies examined the accuracy of 9 ML techniques. The pooled accuracy of the 9 ML techniques was 96% (95% CI 93%-98%; Figure 2). Significant heterogeneity was shown in the meta-analyzed studies (*I*^2^=93.51%; *P*<.001), and the possible causes of this heterogeneity are discussed below. Regarding voice disorders assessment, the ML technique that achieved the highest accuracy was LS-SVM (99%), whereas the one that had the lowest accuracy was QDA (91%).

#### Sensitivity

**Figure 3:**
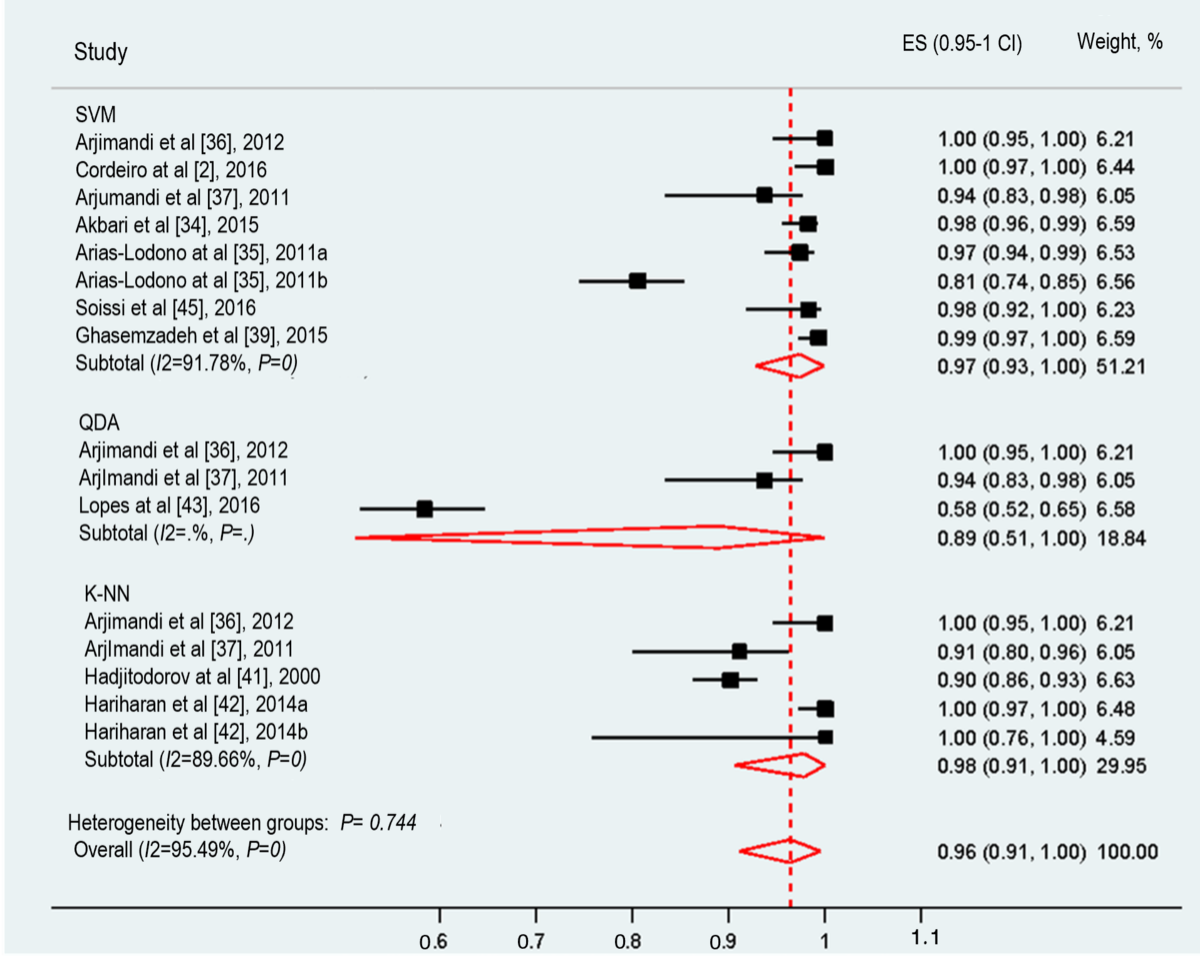
The forest plot shows the sensitivity of machine learning algorithms in voice disorder screening. K-NN: K-nearest neighbor; QDA: quadratic discriminant analysis; SVM: support vector machine.

The sensitivity of ML techniques in assessing voice disorders was reported in 77% (10/13) of studies. These studies examined the sensitivity of 3 ML techniques. The pooled sensitivity of the 3 ML techniques was 96% (95% CI 91%-100%; Figure 3). The meta-analyzed studies showed significant heterogeneity (*I*^2^=95.49%; *P*<.001), and the possible causes of such heterogeneity are discussed in further sections. K-NN had the highest sensitivity (98%) among the 3 ML techniques, while QDA achieved the lowest sensitivity (89%).

#### Specificity

**Figure 4:**
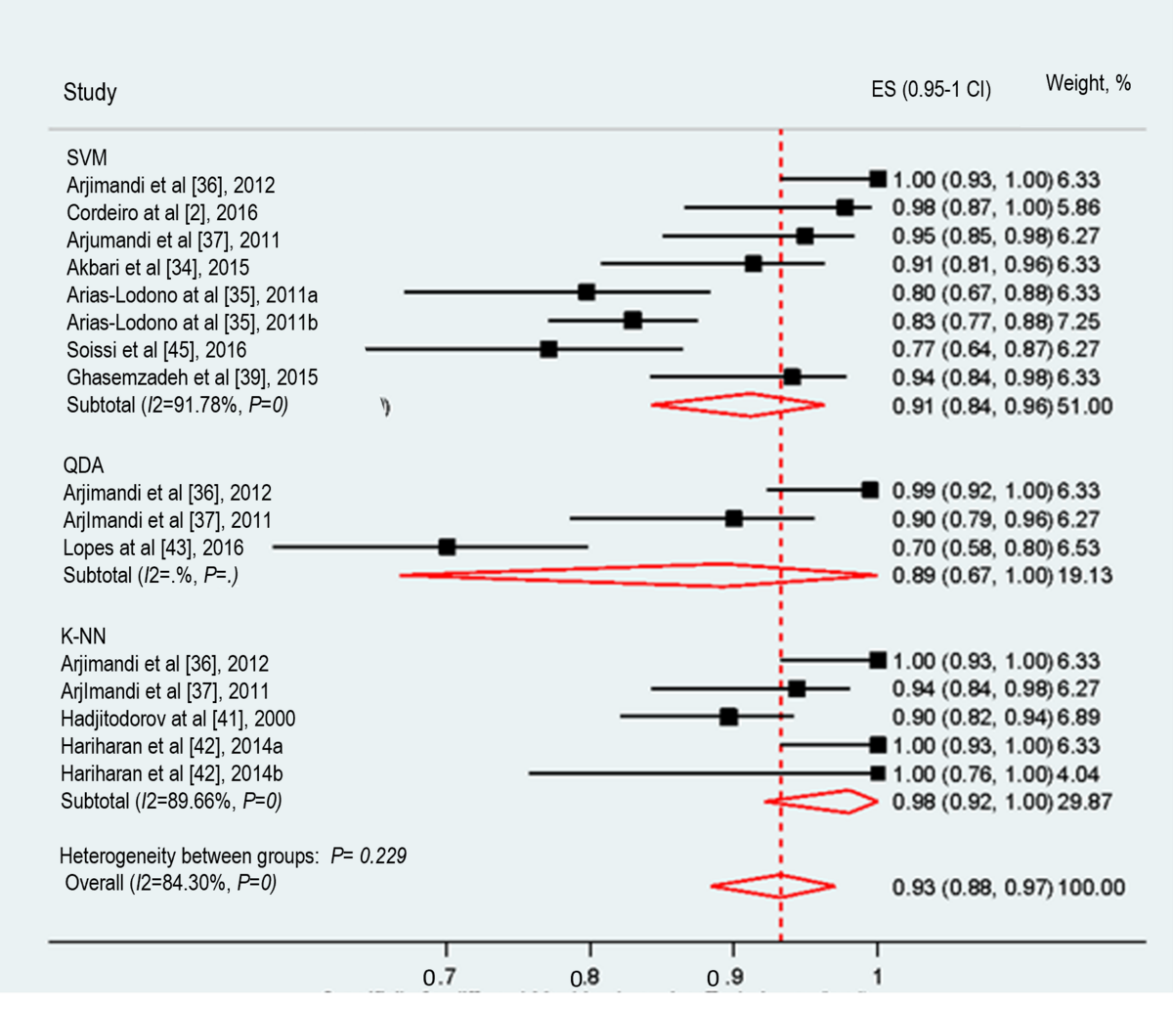
The forest plot shows the specificity of machine learning algorithms in voice disorder screening. K-NN: K-nearest neighbor; QDA: quadratic discriminant analysis; SVM: support vector machine.

The specificity of ML techniques in assessing voice disorders was examined in 77% (10/13) of studies and included the specificity of 3 ML techniques. The pooled specificity of the 3 ML techniques was 93% (95% CI 88%-97%; Figure 4). The meta-analyzed evidence showed significant heterogeneity (*I*^2^=84.3%; *P*<.001); the possible causes of heterogeneity are discussed below. The ML technique that achieved the highest specificity was K-NN (98%), whereas the one that had the lowest specificity was QDA (89%).

#### Heterogeneity and Pooled Performance

The possible source of heterogeneity in the pooled performance was explored, and the possibility that studies that used short-term parameters, such as the study by Arjmandi et al [37], increased the heterogeneity in K-NN and LS-SVM was found. In the K-NN algorithm, the heterogeneity was reduced to 69.73% when the study by Arjmandi et al [37] (which used long-term parameters) was excluded; in specificity, it was 84.92% in sensitivity and 91.55% in accuracy. This was also found in the study by Hadjitodorov et al [41], which also used long-term parameters, and when it was excluded, the heterogeneity in all K-NN outcomes was reduced (Multimedia Appendix 12 presents further details on the heterogeneity values when each study was removed). Similarly, when the study by Arjmandi et al [37] was removed from the LS-SVM forest plot for sensitivity, a reduction was found in *I*^2^ test values, which decreased to 91.89%. Therefore, long-term parameters could affect the sensitivity of LS-SVM and all 3 outcomes in K-NN. Furthermore, the database used by Arias-Londoño et al [35] and Souissi and Cherif [45] might increase the heterogeneity in LS-SVM performance. Arias-Londoño et al [35] used the UPM database, which is a Spanish sounds database, thus excluding the study from the sensitivity and specificity forest plot of LS-SVM, which decreased the heterogeneity to 58% and 71%, respectively. On the other hand, Souissi and Cherif [45] used voice samples from S (German speech samples database), whereas the remaining studies used s from the English speech samples database (MEEI; Multimedia Appendix 12).

## Discussion

### Principal Findings

This study systematically reviewed the performance of ML in assessing voice disorders, similar to another study by Syed et al [27] that examined the accuracy of ML algorithms at the voice database level and qualitatively analyzed the accuracy of each ML algorithm technique. It was concluded that LS-SVM is the most common algorithm used in studies included in this research, which aligns with our findings. Furthermore, the performance showed the accuracy of LS-SVM to be >93%, which was similar to our findings. Generally, ML performance was found to be more promising when it was used as a screening tool rather than in diagnosis, achieving >90% in all 3 outcomes (accuracy, sensitivity, and specificity). Second, the findings differ significantly between the algorithms or even within the same algorithm in different studies. For example, LS-SVM was almost 100% in all 3 outcomes; however, Parzen classifier showed sensitivity ranging from 74% to 100%. Because of the limited number of studies, the performance of ML in ≤2 studies remains unclear. This was also noticed in ML algorithms that were used in the diagnosis, as only 1 study implemented ML algorithms to differentiate between different disorders (diagnosis). For example, the performance of QDA in screening showed 83% accuracy, 91% sensitivity, and 68% specificity. By contrast, it was found to be <76% in diagnosis, and the percentage fell sharply in sensitivity and specificity in the same study [43]. However, this finding could not be conclusive because of the limited number of studies that used ML for diagnosis (1 study).

The analysis implies that K-NN and LS-SVM showed the highest accuracy. K-NN demonstrated increased specificity; however, LS-SVM was found to be better at detecting true positive cases. Because ML in the included studies was used as a screening tool (pathological voice vs healthy voice), the ability of ML to be more sensitive might be more important than the ability to be specific. This may be due to the consequences of diagnosing healthy voiced patients as pathological voice which will only lead to further examination (stroboscopy). Moreover, it will not cause any distress to the patient, as the diagnosis is not final, and patients would only be referred for further examination. However, in less sensitive tests, misdiagnosis of patients can lead to harmful consequences.

### Research and Practical Implications

#### Practical Implications

When a person’s strength, agility, and structure of vocal folds result in pathological noise and reduced acoustic tone, their vocal pathology may be serious enough to qualify as a voice disorder. These disorders can be caused by tissue diseases and changes in tissue, mechanical stress, surface discomfort, systemic changes, changes in muscles and nerves, and many other factors [48]. Research on has achieved a wide scope, partly because of its societal benefits. Standard databases have been developed to mitigate disorders and include new features and emphasis on specific voice disorders while using deep neural networks. Recently, subjective and objective evaluations of vocal issues have received considerable attention in the research field [49].

Subjective assessments may be conducted by clinicians, as they focus on the patient’s voice and use different instruments to discern various vocal disorder diagnoses. ML can be used as a decision-support tool for clinicians conducting auditory-perceptual assessments [14]. A second assessment, known as “target evaluated assessments,” focuses on the automatic, computer-based processing of acoustic signals. These signals assess and recognize the underlying vocal pathology, which may not be screened or diagnosed by a clinician [50]. Consequently, this type of evaluation is nonsubjective. Furthermore, when using this type of assessment, voices can be captured and stored at a global level via cloud technologies by using various intelligent devices. This has been beneficial for researchers across the globe, who can access the data through different academic institutions.

Using ML as an assessment tool may reduce the learning gap between experienced and inexperienced clinicians. Bassich and Ludlow [20] found that the intrajudge test-retest agreement was <75% when evaluating voice quality in patients with polyps or vocal fold nodules; thus, the overall reliance on experienced clinicians in voice assessment might be eliminated. Furthermore, the practice of using instrumental assessments in practice could be eliminated, as ML may reduce the need to conduct instrumental assessments for more typical cases [27]. However, eliminating instrumental assessments altogether may lead to misdiagnoses, for example, if a patient with laryngeal cancer was screened “as healthy,” the clinician may not have performed a stroboscopic examination. Therefore, we aim to further our study by establishing an ideal and automatic ML-based system. We anticipate that this system will be sensitive, accurate, efficient, and successful in detecting and diagnosing various voice disorders quickly and effortlessly for both patients and practitioners.

The review showed that ML provided optimum performance in screening and diagnosing voice disorders to inform clinicians of anomalies. A comparison of the performance of ML algorithms, including accuracy, specificity, and sensitivity, across studies is recommended owing to the different characteristics of each study. The most commonly used ML methods for diagnosing voice disorders in this review were LS-SVM and artificial neural network algorithms. However, the preference of applying 1 ML method to another was not clearly explained in the studies. All studies used internal validation (training and test splits and cross-validation) to evaluate the ML quality. However, external validation is a necessary procedure to evaluate the real quality of ML predictions for new data. Therefore, external validation is essential to implement ML in routine clinical practice to diagnose voice disorders. Therefore, external validation must be performed before using ML for any clinical diagnosis. None of the ML methods investigated in this review used external validation.

#### Implications for Research

This paper analyzes the literature related to the effectiveness of using ML algorithms to screen and diagnose voice disorders. It not only provides insight into the type of research conducted over the last 2 decades but also highlights the areas of research needing further experimentation and analysis. Researchers and practitioners can use this research to improve their objective screening or diagnosis of speech pathology. For instances, voice disorders [23], MEEI [22], and UPM databases [51] are all accessible to researchers interested in voice disorders case studies. However, these data repositories are not without their flaws. For instance, certain databases are uniformly classified into healthy and unhealthy classes. These voices are, in turn, generally categorized as “healthy” and “pathological” in most of the research published using these data. Some databases do not specify the severity of voice disorders or provide sufficient details on the pathological symptoms during phonation. As such, some samples may appear healthy normal despite being labeled as pathological, and vice versa. In addition, >1 disorder may be used to label documents, which can be challenging to incorporate or exclude samples in different languages [52]. The nature of supervised ML, that is, “labeled,” tests require prior knowledge of the reference standard finding to the corresponding test. This may lead to a higher risk of bias in some quality assessment tools, such as the QUADAS-2 tool, which shows a high risk of bias in the index test domain. Future researchers may wish to consider providing information on how a reference standard was applied when examining the performance of ML. Furthermore, these repositories may determine a more specific judgment on suitable demographic characteristics and how to appropriately classify these specifics. Finally, differential diagnostic abilities for ML may be better examined by dividing both the outcomes of each disorder as well as their severity. This would allow for more definitive and specific findings about the type of patients for whom ML may be more effectively used.

Because ML in the included studies was used as a screening tool (pathological voice vs healthy voice), the ability of ML to be more sensitive might be more important than its ability to be more specific. This may be due to the consequences of diagnosing healthy patients as unhealthy (patients with pathological voice), which will lead to further examination (stroboscopy) and not cause patient distress, as the diagnosis, at this point, is not final and patients would be referred for further examinations. Misdiagnosing patients (less sensitive tests) could lead to harmful consequences and distress, for example, if life-threatening diseases such as laryngeal cancer are misdiagnosed.

It should also be considered that ML can be used as a decision-support tool by clinicians while subjectively judging patients’ voices to determine whether they should undergo further examinations. Applying the ML algorithm as a screening tool could help in predetermining the patient’s voice condition. Consequently, this could support the clinicians’ whole management process in voice disorders assessment, especially in their decision on whether to apply an instrumental examination for the patient, a decision that is currently being made subjectively. Therefore, applying ML as a screening tool would reduce the gap between experienced and inexperienced clinicians (the agreement was found to be <75%) [20], and the overall reliance on experienced clinicians in voice assessment might be eliminated. Furthermore, the use of instrumental assessments in practice could be eliminated, as not all patients will have to undergo instrumental assessments (ML might reduce the need to use them for healthy cases). Therefore, the cost of assessing voice disorders might be reduced.

Our findings also imply that ML can be used in web-based methods to detect voice disorders. This means that the algorithms can be used in smartphone apps or users’ phone calls to detect the presence of voice disorders or even track the progress of their therapy. This might eliminate the amount of time spent by the clinician to screen or diagnose or record the progress of each follow-up. This study also found that researchers may want to consider investigating the applicability of various ML algorithms to identify and diagnose voice disorders. moreover, adding to previously established databases is recommended, which includes adding different languages, such as the Arabic voice pathology database, to other mainstream repositories.

### Strengths

The key strength of this review is that it follows the DTA systematic review and search strategy. First, this review was in accordance with the Cochrane Library DTA systematic reviews, and second, it used a variety of medical, computer, and engineering databases. This increased the sensitivity of the review and broadened the search, overcoming the limited number of related articles. Moreover, in the screening process, in cases where the relevance of the abstract was not clear, the study was included in the full-text scanning. This eliminated any chance of eliminating relevant articles from the review. In addition, in the reference standard test, the inclusion criteria were restricted to a controlled environment, which might have ensured a more accurate and reliable result.

This is the first review to systematically assess the performance of different ML algorithms in the assessment and diagnosis of voice disorders. A total of 13 observational studies were included, which recruited patients from both genders and different age groups (13-85 years). In all, 14 ML techniques were tested, 9 of which were included in the meta-analysis, and their pooled accuracies, sensitivities, and specificities were estimated.

### Limitations

The main weakness of this review is the limited reporting by primary studies; for example, the criteria for selecting voice samples from the databases or the patient recruitment process, the poor reporting of the demographic characteristics of the sample, and the severity of the voice disorders in each case. This hindered the ability to find sources of heterogeneity, as subgroup analysis based on gender, age group or type, or severity of each disease could not be investigated. Furthermore, the main outcomes of the review could not be more specific to a certain gender or age group or the type or severity of the disease. Mentioning these details could have allowed for further investigation of which factors—voice disorders, gender, or age group—would determine the accuracy of ML performance. In the patient selection domain, more than half (8/13, 60%) of the included studies demonstrated an unclear risk of bias. The poor reporting of how voice samples were chosen from the database led to the estimated accuracy being subject to bias. The bias increased when the voice samples were not chosen randomly, as they might have been chosen based on unreported severities. However, removing these studies from the meta-analysis was not possible owing to the limited number of included studies.

All included studies (13/13, 100%) failed to report how the reference standard was used, thus leading to an “unclear” risk of bias assessment in the overall reference standard. This is mainly due to the use of voice samples from a database; therefore, the clinicians’ assessment was not performed by the authors of the primary studies. Moreover, the clinicians’ assessment, which was applied by the chosen database, was not reported in the studies. Not knowing how the assessment was performed increased the risk of bias, and the outcome of the review was found to be unclear. Although the authors were contacted to request further details about the choice of voice samples and reference standard assessment, no response was received. Poor reporting led to an unclear risk of bias in the flow and timing of patients in almost all included studies (12/13, 92%), especially the lack of reporting of the time intervals between clinicians’ assessment and the recording of patients’ voices. For example, if the recordings were made at intervals of a few months after the clinician’s assessment, the patients’ condition could have changed from when the first recording was made. Consequently, this increased the chance of misclassification or misdiagnosis, as the voice sample diagnosis could be different from the clinician’s diagnosis. Better reporting of patients’ diagnosis and recruitment process would lead to a clearer risk of bias assessment.

### Conclusions

ML showed promising findings in screening, as its accuracy, sensitivity, and specificity showed high performance. The findings also suggested that ML can be further used in new smartphone apps for screening purposes and that screening can be conducted on the web. In scholarly research, more research with specific patient demographics and disorders is recommended. However, definitive conclusions could not be drawn about the effectiveness of ML in diagnosing owing to the limited number of studies (only 1). Therefore, we recommend using ML as a decision-support tool for clinicians during screening. For more definitive conclusions regarding the use of ML in diagnosis, more studies are suggested to be conducted, and risk of bias assessment that suits the application of ML for medical purposes and supervised ML is encouraged.

## Appendix

Multimedia Appendix 1 Search strategy in Web of Science.

Multimedia Appendix 2 Search strategy in MEDLINE.

Multimedia Appendix 3 Extraction table.

Multimedia Appendix 4 Quality Assessment of Diagnostic Accuracy Studies 2 tool; patient selection domain.

Multimedia Appendix 5 Quality Assessment of Diagnostic Accuracy Studies 2 tool; index test domain.

Multimedia Appendix 6 Quality Assessment of Diagnostic Accuracy Studies 2 tool; reference standard domain.

Multimedia Appendix 7 Quality Assessment of Diagnostic Accuracy Studies 2 tool; flow and timing domain.

Multimedia Appendix 8 Modified Quality Assessment of Diagnostic Accuracy Studies 2 tool for this systematic review.

Multimedia Appendix 9 Quality Assessment of Diagnostic Accuracy Studies 2 tool risk of bias judgment in each included study across all domains and applicability concerns.

Multimedia Appendix 10 Summary of the performance of machine learning algorithms that were used for diagnosis.

Multimedia Appendix 11 Summary of the performance of machine learning algorithms that were used in screening.

Multimedia Appendix 12 The heterogeneity values after removing heterogenous studies from each forest plot.

## Abbreviations

DTA: diagnostic test accuracy
K-NN: K-nearest neighbor
LS-SVM: least-squares support-vector machine
MEEI: Massachusetts Eye and Ear Infirmary
ML: machine learning
QDA: quadratic discriminant analysis
QUADAS: Quality Assessment of Diagnostic Accuracy Studies
SLT: speech and language therapist
UPM: Universidad Autónoma de Madrid
