# Supplementary figures and images for: The Effectiveness of Supervised Machine Learning in Screening and Diagnosing Voice Disorders: Systematic Review and Meta-analysis
Source: J Med Internet Res. 2022 Oct 14;24(10):e38472. doi: 10.2196/38472 (PMC9617188; doi:10.2196/38472)

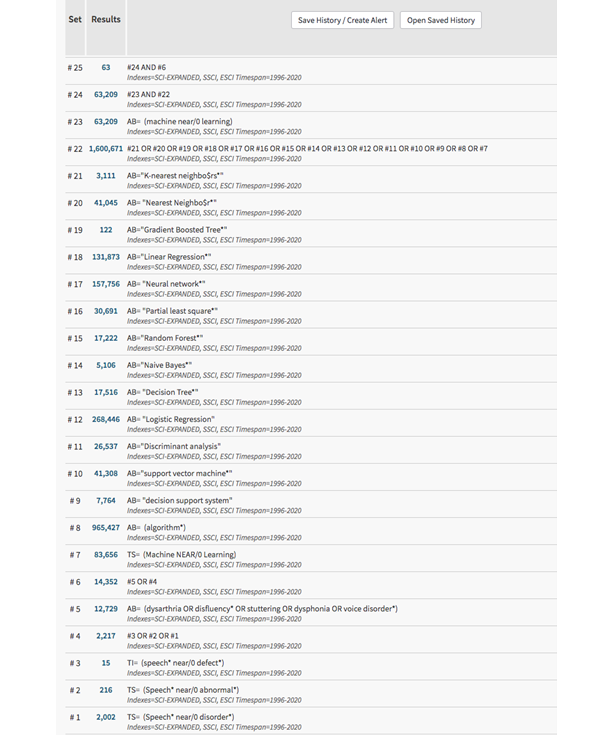

Supplement: Multimedia Appendix 1 [file jmir_v24i10e38472_app1.png]

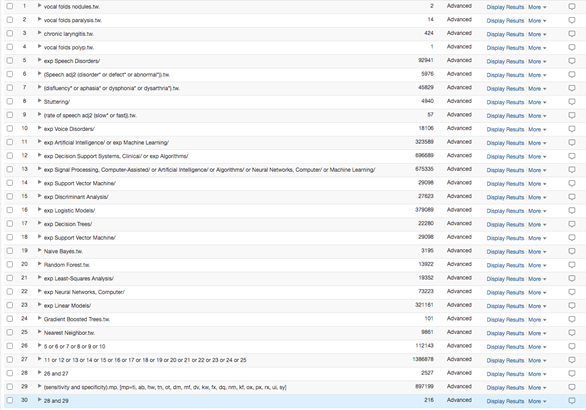

Supplement: Multimedia Appendix 2 [file jmir_v24i10e38472_app2.png]

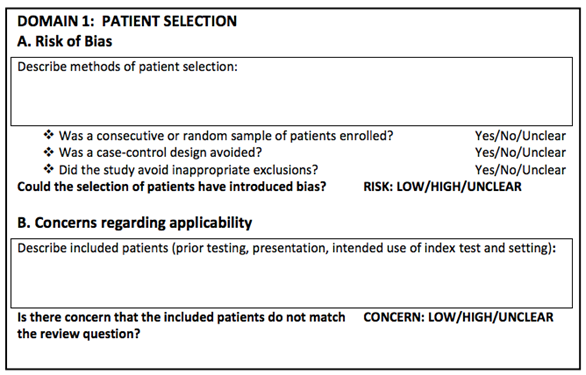

Supplement: Multimedia Appendix 4 [file jmir_v24i10e38472_app4.png]

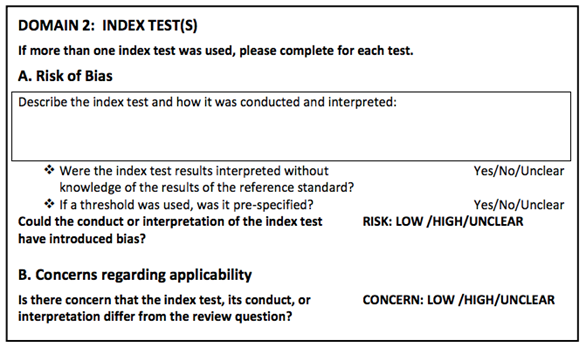

Supplement: Multimedia Appendix 5 [file jmir_v24i10e38472_app5.png]

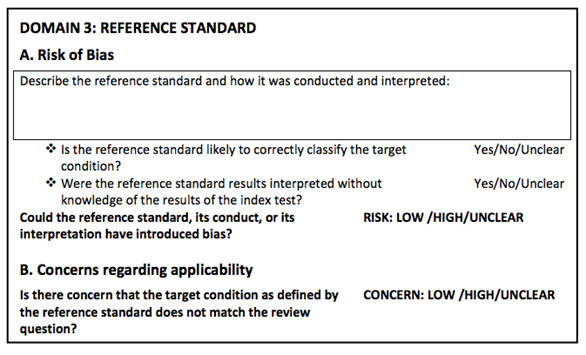

Supplement: Multimedia Appendix 6 [file jmir_v24i10e38472_app6.png]

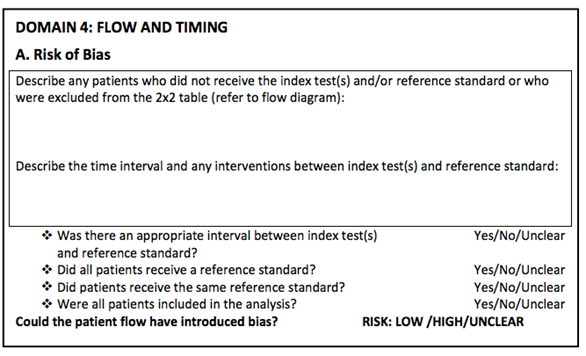

Supplement: Multimedia Appendix 7 [file jmir_v24i10e38472_app7.png]

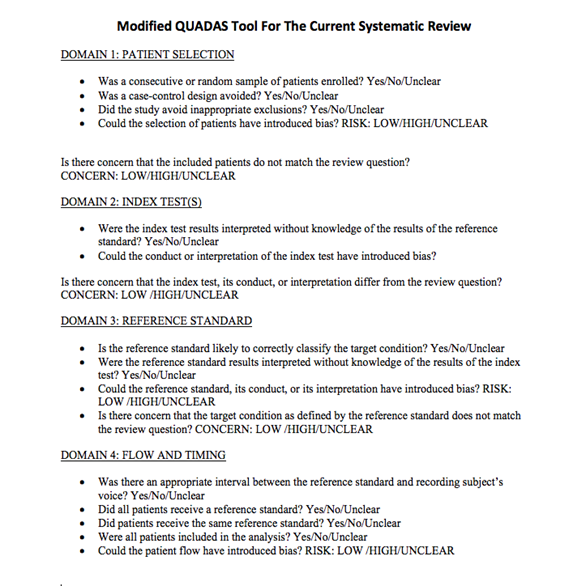

Supplement: Multimedia Appendix 8 [file jmir_v24i10e38472_app8.png]

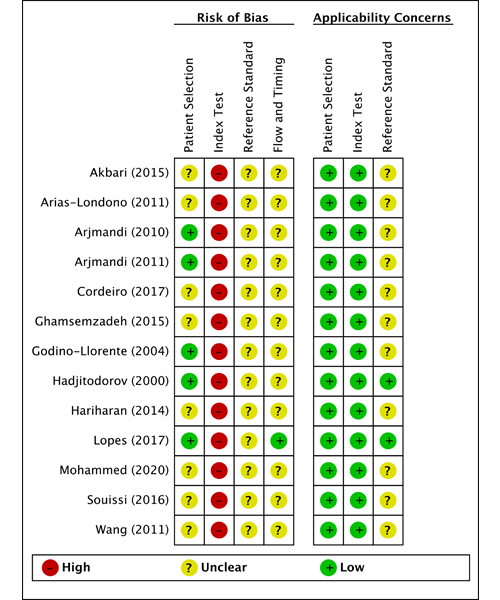

Supplement: Multimedia Appendix 9 [file jmir_v24i10e38472_app9.png]

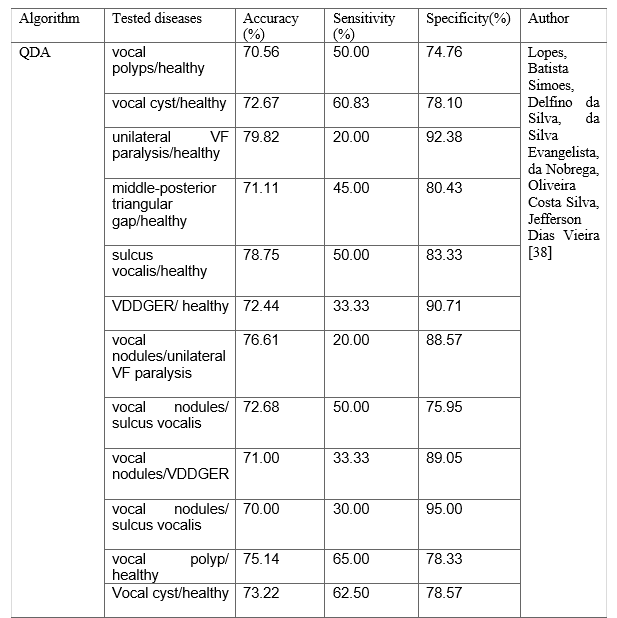

Supplement: Multimedia Appendix 10 [file jmir_v24i10e38472_app10.png]

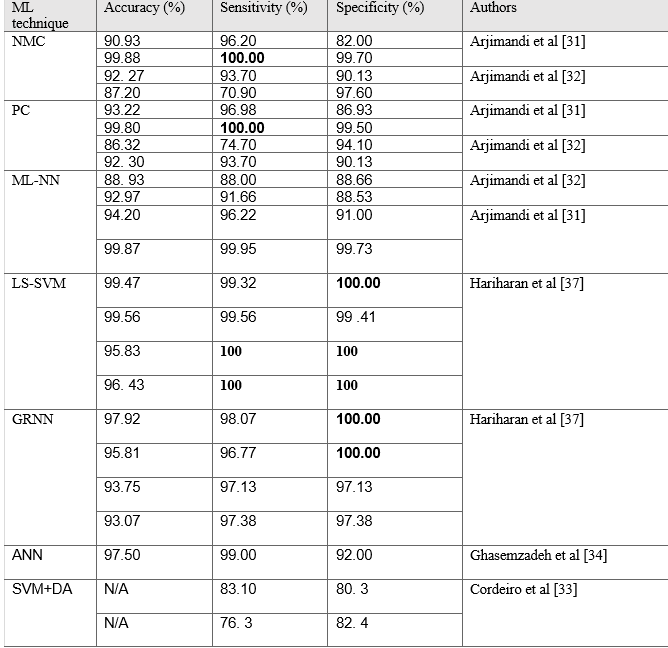

Supplement: Multimedia Appendix 11 [file jmir_v24i10e38472_app11.png]

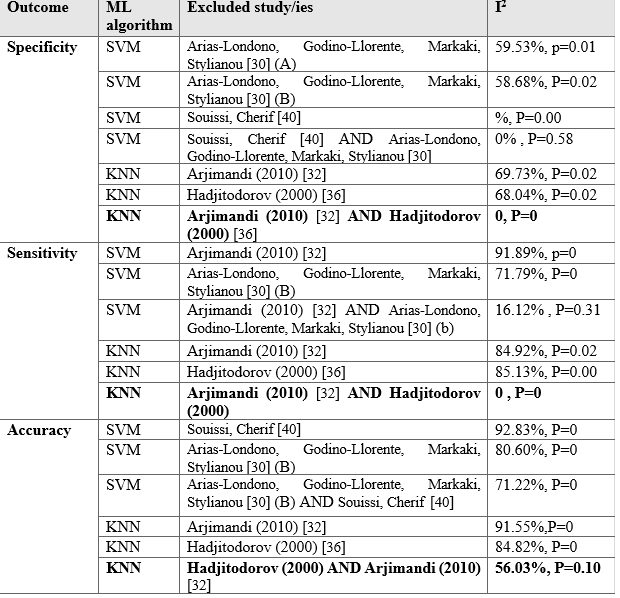

Supplement: Multimedia Appendix 12 [file jmir_v24i10e38472_app12.png]
