# Supplementary material for: The Effectiveness of Supervised Machine Learning in Screening and Diagnosing Voice Disorders: Systematic Review and Meta-analysis
Source: J Med Internet Res. 2022 Oct 14;24(10):e38472. doi: 10.2196/38472 (PMC9617188; doi:10.2196/38472)
Supplement: Multimedia Appendix 3 [file jmir_v24i10e38472_app3.docx]

Appendix 3. Table of Extraction Form
